# *Chlamydia trachomatis* Antibody Testing in Vaginal Mucosal Material versus Blood Samples of Women Attending a Fertility Clinic and an STI Clinic

**DOI:** 10.1155/2014/601932

**Published:** 2014-03-13

**Authors:** Ingrid V. F. van den Broek, Jolande A. Land, Jan E. A. M. van Bergen, Servaas A. Morré, Marianne A. B. van der Sande

**Affiliations:** ^1^Epidemiology & Surveillance Unit, Centre for Infectious Disease Control, National Institute of Public Health and the Environment (RIVM), P.O. Box 1 (75), 3720 BA Bilthoven, The Netherlands; ^2^Department of Obstetrics and Gynaecology, University Medical Center Groningen, University of Groningen, P.O. Box 30.001, 9700 RB Groningen, The Netherlands; ^3^STI AIDS Netherlands (SOA AIDS Nederland), P.O. Box 10845, 1001 EV Amsterdam, The Netherlands; ^4^Department of General Practice, AMC-UVA, P.O. Box 22660, 1100 DD Amsterdam, The Netherlands; ^5^Laboratory of Immunogenetics, Department of Medical Microbiology and Infection Control, VU University Medical Centre, De Boelelaan 1105, 1081 HV Amsterdam, The Netherlands; ^6^Institute of Public Health Genomics, Department of Genetics and Cell Biology, Research Institutes CAPHRI and GROW, Faculty of Health, Medicine & Life Sciences, University of Maastricht, P.O. Box 616, 6200 MD Maastricht, The Netherlands; ^7^Julius Centre UMCU, P.O. Box 85500, 3508 GA Utrecht, The Netherlands

## Abstract

*Background.* Chlamydia infections often follow an asymptomatic course but may damage the reproductive tract. Chlamydia antibodies in serum are used as markers for past infections and can relate to tubal pathology and infertility. This *“proof of principle”* study aimed to assess whether Chlamydia antibodies are detectable in easier to obtain, noninvasive, vaginal mucosa samples and relate to current or past infection. *Methods.* We compared outcomes of Chlamydia IgG and IgA antibody tests in serum and vaginal mucosal swabs in (a) 77 women attending a fertility clinic, of whom 25 tested positive for serum-IgG and (b) 107 women visiting an STI centre, including 30 Chlamydia PCR-positive subjects. *Results.* In the STI clinic, active Chlamydia infections were linked to serum-IgG and serum-IgA (*P* < 0.001) and mucosa-IgA (*P* < 0.001), but not mucosa-IgG. In the fertility clinic, mucosa-IgG had stronger correlations with serum-IgG (*P* = 0.02) than mucosa-IgA (*P* = 0.06). Women with tubal pathology or Chlamydia history more commonly had serum-IgG and mucosa-IgA (both *P* < 0.001), whereas this link was weaker for mucosa-IgG (*P* = 0.03). *Conclusion.* Chlamydia IgG and IgA are detectable in vaginal mucosal material. Serum-IgG had stronger associations with current or past infections. Mucosa-IgA also showed associations with (past) infection and complications. IgA presence in vaginal mucosa warrants further epidemiological studies.

## 1. Introduction

*Chlamydia trachomatis* is a common sexually transmitted infection among adolescents. In women, lower genital tract infections (cervicitis) may ascend to the upper genital tract and cause pelvic inflammatory disease (PID), potentially leading to tubal pathology and subsequent infertility [1, 2]. Chlamydia cervicitis and PID often remain asymptomatic and PID is difficult to define and diagnose [3], which makes treatment and surveillance of late sequelae difficult. It may take a decade or more before tubal pathology as a cause of infertility becomes apparent, and the invasive and costly surgical procedure of laparoscopy remains the recommended way of diagnosis [4].

Various immunological markers have been studied for their ability to indicate an individual's history of Chlamydia infection and increased probability for late complications [5–8]. In infertile women, the presence of Chlamydia IgG antibodies in serum is associated with tubal pathology and lower natural conception rates, even in case of tubal patency [9]. Elevated levels of anti-Chlamydia antibodies can be detected in 30–70% of women with tubal pathology [5, 10–12] compared to around 10–20% in the general female population of reproductive age [13, 14]. In fertility clinics in the Netherlands, Chlamydia IgG antibody testing (IgG-CAT) in serum is used as a screening test for tubal pathology and for selecting high-risk patients for laparoscopy [15, 16]. A more proximal, noninvasive biomarker enabling selection of women at high risk of late complications of Chlamydia infection would be useful for targeted prevention at the individual level but also facilitate natural history studies and provide an outcome marker for screening intervention studies and candidate vaccine trials [17]. Furthermore, a biomarker in vaginal material collected by (self-) swab indicating that increased risk of previous Chlamydia infection, PID or, tubal pathology could be of high value not only for identifying cases but also for identifying “controls,” for example, in a population-based study. The presence of Chlamydia antibodies in vaginal or cervical samples has not been studied extensively in this context before, although results of an early study by Brunham et al. [18] and Agrawal et al. [19] and unpublished data (Morré, personal communication) have shown that IgA can be detected in cervical swab material and in women with a current Chlamydia infection. It is yet unknown whether the IgG or IgA CAT assay can be applied in (self-collected) vaginal swabs of mucosa instead of serum samples. The advantage would be that this sampling method is less invasive and vaginal samples are available from all women at the time of a regular Chlamydia (PCR) test.

The further aim of the current “*proof of principle*” study was to assess the usefulness of the application of the CAT-test on mucosal swabs for identification of women who have or have had an infiltrating Chlamydia infection that could be the cause of late complications. We hypothesized that local, mucosal IgG antibodies can persist in the lower genital tract mucosa for a long time (similar to IgG in serum), while IgA antibodies may disappear in the course of time, provided that the infection is cleared. IgA antibodies in serum are assumed to reflect chronic inflammation and have been shown to be useful in the serodiagnosis of tubal factor infertility caused by* C. trachomatis *[6, 12]. Anti-Chlamydia IgA in serum has also been linked to another complication of Chlamydia, that is, LGV [20, 21]. Hence, IgA most likely indicates an active, on-going, persistent infection, rather than a past, cleared infection, which makes it a potential candidate for the biomarker we are looking for.

In this study, we compared (A) the correlation between the outcomes of the serum-IgG CAT and the mucosa-CAT in a group of women at risk for late complications (attending a fertility clinic) and (B) the correlation between the presence of Chlamydia DNA (PCR) in vaginal swabs and the outcomes of the serum-CAT and mucosa-CAT (IgG and IgA), in women at risk for a current infection (attending an STI clinic).

## 2. Methods

### 2.1. Study Population and Recruitment

We performed a comparative diagnostic study, in two groups of patients.

**Figure 1:**
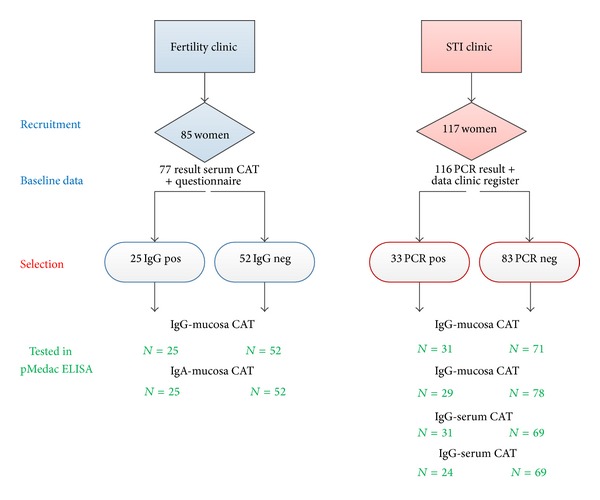
Flowchart showing the number of patients selected during recruitment and the number of different CAT tests performed in the two patient groups.

*(A) Women attending the fertility clinic* of the University Medical Center in Groningen (UMCG) in January and February 2012: healthy, 20 to 40-year-old women, who had a serum IgG CAT (CT pELISA, Medac, Wedel, Germany) taken within the previous year(s) as part of their fertility workup received a written request to participate. After consent was given, they received a short questionnaire on past Chlamydia infections or PID and a test-kit with a vaginal swab for self-collection. Further relevant data were obtained from the medical records at a later stage in the clinical investigations. Recruitment continued until a number of 25 serum-CAT-positive and 50 serum-CAT-negative women was reached. In total, 85 agreed to participate and for 79 of them, complete questionnaire data and samples were obtained (93%), while results of serological CAT were available for 77 women (from current or previous clinic visits). Of the 77 women, 52 had serum-CAT-negative results and 25 serum-CAT-positive results for Chlamydia IgG antibodies, in accordance with the sampling plan. Mucosa-CAT results (IgG and IgA) were obtained for all 79 women who returned the swab (see Figure 1 for details on patient inclusion). The Research Ethics Board of the UMCG approved this study and all women provided informed written consent.

*(B) Women attending the sexually transmitted infection (STI) clinic* in The Hague between September 2011 and February 2012: this study population consisted of women of all ages, who were tested for Chlamydia DNA as part of their STI consultation and consented to the use of their (anonymously stored) remaining sample material from serum and swab for further STI-related research. During consultation, standard information on the person's background and sexual behaviour and risk factors was collected and entered into an electronic patient database for STI surveillance. Recruitment continued until results for 30 PCR-positive and 70 PCR-negative women were obtained. In total, sample material from 117 women was analysed. Results of swabs were available for 115 women and serum results for 102 women; one woman with serum results only was excluded from analysis. Of the remaining 116 women, 33 were PCR positive for Chlamydia and 83 PCR-negative (see Figure 1 for details on numbers included and tested). The laboratory data for all women were linkable to anonymized data collected during consultation in the electronic database by a unique code. Studies using anonymized patient samples from STI clinics are exempted from additional ethical review (VUMC Research Ethics Board for STI Centres in the Netherlands); patients are informed by poster/leaflet in the clinic that material may be used for research routinely unless they object.

### 2.2. Chlamydia Antibodies

The main outcome parameters were presence of IgG and IgA Chlamydia antibodies in vaginal swabs and in serum. Already available results of serum-CAT were used for women from the fertility clinic (IgG only). Serum samples and material collected by vaginal swabs were analysed with Medac Ct IgG/IgA ELISA test (CT pELISA; Medac, Wedel, Germany) in the Laboratory of Immunogenetics, VU University Amsterdam. Tests were performed according to manufacturers' instructions. This Medac test uses a species-specific synthetic peptide based on the major outer membrane protein (MOMP) as an antigen, ensuring minimum cross-reactivity with Chlamydia pneumonia [22, 23]. Performance comparison of the ELISA test to a micro-immunofluorescence assay found the sensitivity and specificity of the CAT test to be 71.4 and 97.3, respectively [22].

### 2.3. Analyses

The qualitative results of serum- and mucosa-CAT were compared with each other and with PCR results and proportions with positive IgG/IgA calculated. At the STI clinic, some test results remained “equivocal” (optical densities between the values for negativity and positivity), because the samples could not be submitted to a confirmation test (1% of serum IgG results, 8% of serum IgA, 11% of swab IgG, and 7% of swab IgA); these samples were excluded from further calculations.

In order to evaluate the agreement of the mucosa-CAT results to the outcomes of PCR and serum-CAT, we calculated Kappa-values for mucosa IgG and IgA versus PCR (in the STI clinic only) and versus IgG in serum (both for the fertility and the STI clinic). We searched for potential relations between IgG or IgA CAT-test outcomes and (determinants of) current and past Chlamydia infection and sequelae thereof (Chi-square, univariate and multivariate logistic regression). In the STI clinic, the question combined history of Chlamydia, gonorrhoea, or syphilis diagnosis in the past 2 years, but in this setting and in the age group of heterosexual women, such a previous STI is most likely to be a Chlamydia episode [24]. All analyses were performed with IBM SPSS Statistics (version 19.0).

## 3. Results

### 3.1. Population Characteristics

The women in the fertility clinic had a median age of 32 years (IQR 28–36 yrs.) at the time of the questionnaire. Fifty-four of 79 (68%) women reported to have been tested for STI in the past and 13 of these 54 (24%) reported a history of Chlamydia; one woman reported a past PID. Clinical data from the women's medical records (6 months later) showed that nine women were diagnosed with tubal pathology and four had experienced an extrauterine pregnancy, while 12 women had become pregnant spontaneously and 56 women had no tubal damage-related diagnosis (11 underwent laparoscopy and 27 hysterosalpingography, 18 no further diagnostics).

The women at the STI clinic had a median age of 22 years (IQR 22–25 yrs.). The majority (113/116) had heterosexual relationships and three were bi-/homosexual; 40 of 116 (34%) reported to have had one partner in the last 6 months, 28 (24%) had 2 partners, and 47 (41%) had three partners or more; 106/116 (91%) had a Dutch background and 10 had non-Dutch background. As a reason for their visit, 24 (21%) mentioned STI-related complaints and 11 (9.5%) were notified to get tested by a sex-partner. Among 33 Chlamydia positives, two had a gonorrhoea infection as well.

### 3.2. Outcomes of the Chlamydia-Antibody Tests in Serum and Swab

In the group of women recruited in the STI clinic, IgG was detected in 36% and IgA in 14% of the sera, while in the mucosal swabs this was 39% (IgG) and 19% (IgA). In the selected group of women in the fertility clinic, IgG was detected in 44% and IgA in 10% of the mucosal swabs; IgG in serum was reported in 32% of women (selection aimed to include 25 positives).

### 3.3. Comparative Analysis Serum-Swab IgG-IgA

**Figure 2:**
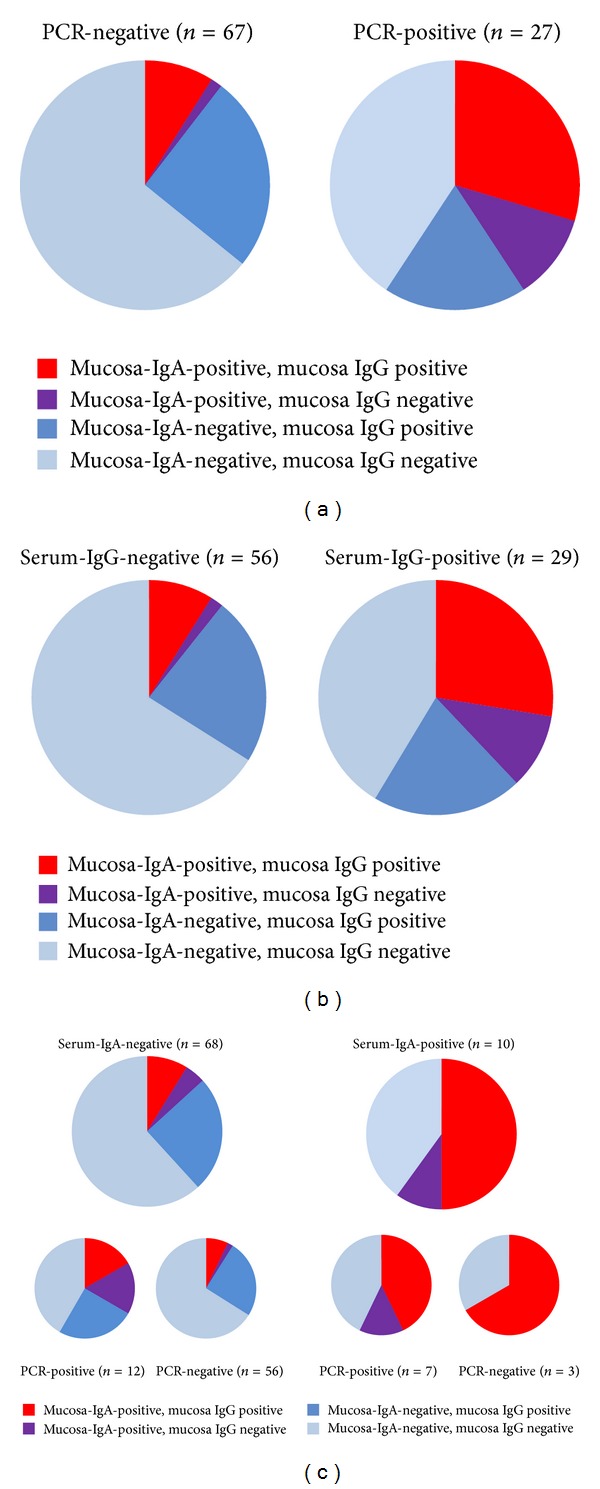
Proportion of samples with detectable chlamydia specific IgA and IgG in vaginal mucosal samples from women who tested positive or negative for (a) Chlamydia PCR, (b) Chlamydia IgG in serum, and (c) Chlamydia IgA in serum, at the STI clinic (MHC The Hague, 2011/2012); connected smaller pie charts below show the same proportions for PCR-positive and PCR-negative samples (indicating presence/absence of acute infection).

*(A) Women in the STI Clinic.* Of the women with a current, PCR-confirmed, Chlamydia infection in the STI clinic, the large majority (94%) had IgG antibodies and one-third (38%) had IgA antibodies in the serum, while only 10% of women without infection had IgG and 6% IgA in the serum. The correlation between the result of PCR tests was high for IgG presence in serum (Kappa 0.799, *P* < 0.001) and modest for IgA (Kappa 0.373, *P* < 0.001). The vaginal swab material of PCR-positive women tested positive for IgG in 48% of cases and for IgA in 41% of cases, while these values were 35% and 10% in PCR-negative women (Figure 2(a)); here IgA-presence was a better “marker” for PCR positivity than IgG presence (Kappa IgA 0.345, *P* < 0.001, and IgG 0.122, *P* = 0.21). Comparing the results of serum-CAT and mucosa-CAT (Figure 2(b)) showed that serum-IgG positivity had similar associations with mucosal antibodies as PCR positivity, with Kappa values of 0.336 for IgA (*P* = 0.001) and 0.130 (*P* = 0.2) for IgG.

Women who tested serum-IgA-positive (*n* = 10) often also had IgA antibodies in the swab (6/10); most serum-IgA-negative women also had no IgA in the swab (64/74; 86%, Kappa 0.37, *P* < 0.001). The association between serum-IgA and swab IgA was most visible in PCR-negative women (Figure 2(c)). There was no association between IgA in serum and IgG in swabs (Kappa 0.12, *P* = 0.18).

Women with a history of STI (*n* = 11) had a higher chance to have detectable serum-IgG than women with no prior STI history (73% versus 32%, *P* = 0.022), but their chance to have a mucosa-IgG was lower (20% versus 41%, n.s. *P* = 0.17; see Table 1). IgA presence (serum or swab) was not correlated to STI history. Other factors influencing the outcomes of the antibody tests were presence of physical symptoms, condom use, number of partners in the last 6 months, and ethnicity. Women who came to the STI clinic with physical complaints (*n* = 20) had a higher chance to have a positive IgG in serum than women who had no complaints (55% versus 31%, *P* = 0.045). Similar differences were seen for women who reported not to have used a condom at the last sexual contact (*n* = 31) compared to those who did (26% versus 44%, *P* = 0.044; see Table 1). No relation with IgA in serum or mucosa was found. The number of partners in the last 6 months (in three categories: 0 or 1, 2, and 3 or more) was a determinant for serum-IgA (*P* = 0.019) and borderline for serum-IgG (*P* = 0.06), but not for mucosa-IgG or -IgA presence. Women with a non-Dutch background (*n* = 10), mucosal CAT results were more often positive for IgG (70%) and IgA (60%) than among Dutch women (36% and 14%, *P* = 0.04 and *P* = 0.003), whereas there was no significant difference in the proportion serum-CAT positives.

**Figure 3:**
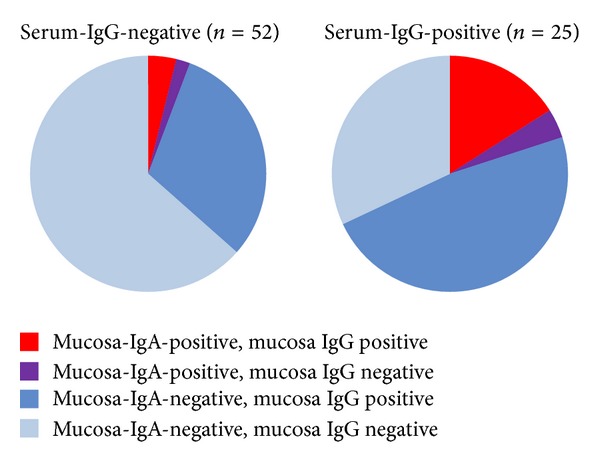
Proportion of samples with detectable chlamydia specific IgA and IgG in vaginal mucosal samples from women who tested positive or negative for Chlamydia IgG in serum, at the fertility clinic (University Medical Centre Groningen, 2012).

*(B) Women in the Fertility Clinic.* Among women with serum-IgG, 64% also had IgG antibodies in the vaginal mucosa, while 35% of the seronegative women tested positive for mucosa-IgG (Figure 2). The Kappa value indicated a modest rate of agreement between systemic and local IgG tests (Kappa 0.27, *P* = 0.015). Mucosa-IgA was found in 20% of the women with serum-IgG and in 6% of the serum-negatives (Figure 3), resulting in a lower rate of agreement (Kappa 0.17; *P* = 0.055).

Women diagnosed with a reported tubal pathology diagnosis (*N* = 9) more often tested positive on serum-IgG (89%) than women without tubal pathology (25%, *P* < 0.001) and more often for mucosa-IgA (44% versus 6%, *P* = 0.005), but not for mucosa-IgG (56% versus 43%). Similarly, women with a history of Chlamydia infection (self-reported or noted in medical records; *n* = 21) more often had serum-IgG and mucosa-IgA and -IgG than those without such a history (serum-IgG: 81 versus 14%, *P* < 0.001; mucosa-IgA: 33 versus 1.7%, *P* < 0.001; mucosa-IgG: 67 versus 36%, *P* = 0.02).

### 3.4. Results from the Two Study Cohorts Together

In samples from women in the STI clinic, Chlamydia infections (determined by PCR) were linked to both serum-IgG (Figure 2) and serum-IgA and to mucosa-IgA, but not to mucosa-IgG. In women in the fertility clinic, IgG in serum was found in most women with tubal pathology and in women reporting a history of Chlamydia. We also found that women with tubal pathology or Chlamydia history more often had mucosal IgA than women with neither of these, whereas this difference was not seen for mucosal IgG. In the STI clinic, mucosa-IgA correlated with serum-IgG in serum. In the group of women recruited at the fertility clinic, mucosa-IgG had a stronger correlation with serum-IgG than with mucosa-IgA.

Altogether, IgG in serum was the best predictor for a current or probable past Chlamydia infection (combining PCR-confirmed current infection with a history of STI) in the STI clinic and also the best predictor for “Chlamydia and related complications” (combining Chlamydia history, PID, and tubal pathology) in the fertility clinic. IgA in swabs showed high specificity for tubal pathology in the fertility clinic and Kappa values were consistently higher for IgA than IgG in swab (see Table 2).

## 4. Discussion

Chlamydia antibody testing can detect local IgG and IgA in vaginal mucosal swab material from women with a current Chlamydia infection or probable exposure in the past. The correlations between local anti-Chlamydia antibodies and systemic antibodies or (past) Chlamydia infection were however not unambiguous and warrant further prospective studies. IgG antibodies in serum were the best predictor of a current or potential past Chlamydia infection, in both groups under study: women at the STI clinic and in the fertility clinic. Serum-IgG results were not exchangeable with the results of antibody tests in vaginal mucosa in a straightforward way; however, mucosa-IgA showed a modest association with (past) Chlamydia infection but a stronger association, also compared to serum-IgG, with tubal pathology as a potential long-term complication thereof. Combining results on systemic and mucosal immunological responses seems promising to get more insights into the pathogenesis of Chlamydia.

It is known that up to 80% of the Chlamydia infections in women are asymptomatic [25], and therefore infections are often not detected and treated. Most infected women seem to have an adequate local immune response to clear the infection within months or years thereafter (45% after one year [26], 94% at 4 years of follow-up [27]), but a small proportion of infected women will have a long-lasting Chlamydia infection. A persistent (silent) Chlamydia infection [28, 29] can cause a chronic low-grade immune response, which might induce destruction of tubal epithelial cells and cause tubal pathology [30]. Systemic antibodies correlate to (past) Chlamydia infections and related complications [5–12]. Little is known about the presence of local Chlamydia antibodies in the cervicovaginal mucosa. The dominant Chlamydia antibody in cervicovaginal fluid is IgG rather than IgA [31]. In women with a current Chlamydia infection, presence of combined Ct IgMAG antibodies was recorded in 83% and IgA in 38% [18]; interestingly IgA showed a clear (inverse) correlation with the number of bacteria detected in the cervical secretions tested. Another study reported a 43% IgG and 42% IgA prevalence in cervical material of women with a current infection whereas both prevalences were 7% in women with no current infection or indication of a past infection (no systemic IgG) [19]. These authors also reported that the mucosal antibody prevalence was higher in women with a primary Chlamydia infection than women with a recurrent infection, and this difference was more obvious for IgG (78% in primary and 23% in recurrent infections) than for IgA (52% versus 37%). In our study, mucosal IgA appeared to have a stronger predictive value than IgG. IgA presence in vaginal mucosa might indicate an ongoing Chlamydia infection or chronic low-grade immune response in the upper genital tract, in women who have negative PCR results of the lower genital tract. To investigate this further, a more longitudinal study on the outcome of IgA positivity in the vaginal mucosa in (a cohort of) women with a known past or recent Chlamydia infection would be required.

Our study was unique in exploring the added value of using mucosal (self) swabs to identify markers for adverse outcomes of Ct infections. On the other hand, as this was a small exploratory study, only preliminary conclusions can be drawn; further research and test validation are recommended. Due to the small and selective sample, the estimation of (adjusted) risk factors (as shown in Table 1) may have been biased. We saw a few women with negative serum-IgG/IgA but positive mucosal IgG/IgA test results or vice versa. We think that these are true “discrepancies” between mucosal and serological samples, indicating that only a local response to the Chlamydia infection was evoked, but in further studies, CAT tests for mucosal material should be validated to confirm that specificity for local antibodies in the mucosa is as good as for serological antibodies. There may be different subtypes of antibodies in serum and in other body fluids (dimeric, “secretory IgG/IgA”, versus serum IgG/IgA or subclasses such as IgG1, IgG2, etc.). There is limited information available regarding IgG subtype and class responses after genital* Chlamydia trachomatis* infections [32]. The goat-anti-human Chlamydia antibodies of the conjugate in the Medac pELISA are considered to detect different subtypes as well as the dimeric form (personal communication with Dr. K. Dreesbach, Medac Germany). Furthermore, cervical IgA and IgG levels may fluctuate during different phases of the menstrual cycle [33, 34]. Although it is not known if this also holds for Chlamydia antibodies and to what extent, this may have influenced the mucosal CAT results of some of the patients in our study (we had no information on the menstrual cycle of the women in study). The representativeness of the patients in our study was limited due to the predetermined selection of one-third per patient group with positive and two-thirds with negative results for the CAT-test (in the fertility clinic) and the PCR (in the STI clinic). The proportion of serum-IgG-positive samples among PCR-positives was higher (94%) than what was previously reported (30–70% [5, 10–12, 35]).

Our data suggest that local IgA antibodies might have a stronger discriminative value than local IgG, as IgA presence in the vaginal mucosa was associated with tubal pathology and history of Chlamydia infection in women in the fertility clinic and it correlated to current Chlamydia infection and to the presence of IgG in serum in the group of women in the STI clinic. Provided that the mucosal CAT test has a high specificity, larger population-based studies and further prospective cohort studies are needed to assess to what extent and when IgA in vaginal (self-)swabs could be of value as a biomarker, on its own or in combination with other immunological host-related markers, indicating which women have a higher chance to develop late sequelae from Chlamydia infections.

## Figures and Tables

**Table 1:** Number and proportion of samples with detectable IgG or IgA Chlamydia antibodies per subgroup, determined in serum and vaginal swab samples from women seen at the STI clinic and at the fertility clinic.

		Serum-IgA		%	Serum-IgG		%	Swab-IgA		%	Swab-IgG		%
		*N*_pos_	*N*_total_		*N*_pos_	*N*_total_		*N*_pos_	*N*_total_		*N*_pos_	*N*_total_	
STI clinic	116	13	93	(14%)	36	100	(36%)	20	107	(19%)	40	102	(39%)
PCR													
+	33	9	24	**(38%)**	29	31	**(94%)**	12	29	**(41%)**	15	31	(48%)
−	83	4	69	**(6%)**	7	69	**(10%)**	8	78	**(10%)**	25	71	(35%)
Ct history													
+	11	1	11	(9%)	8	11	**(73%)**	2	8	(25%)	2	10	(20%)
−	115	12	81	(15%)	28	88	**(32%)**	18	98	(18%)	38	92	(41%)
Symptoms													
+	24	2	17	(12%)	11	20	**(55%)**	4	22	(18%)	6	19	(32%)
−	116	11	76	(14%)	25	80	**(31%)**	16	85	(19%)	34	83	(41%)
Condom use													
+	34	3	28	(11%)	8	31	**(26%)**	6	34	(18%)	8	21	(38%)
−	74	9	59	(15%)	27	62	**(44%)**	14	65	(22%)	31	68	(46%)
Partners < 6 months													
0 or 1	41	0	31	**(0%)**	8	32	(25%)	4	38	(11%)	12	34	(35%)
2	28	5	28	**(18%)**	8	28	(29%)	6	24	(25%)	10	27	(37%)
3 or more	47	8	34	**(24%)**	20	40	(50%)	10	45	(22%)	18	41	(44%)
ethnicities													
Dutch	106	11	84	(13%)	32	92	(35%)	14	97	**(14%)**	33	92	**(36%)**
non-Dutch	10	2	9	(22%)	4	8	(50%)	6	10	**(60%)**	7	10	**(70%)**
Fertility clinic	77	n.a.			25	77	(32%)	8	77	(10%)	34	77	(44%)
Tubal pathology													
+	9				8	9	**(89%)**	4	9	**(44%)**	5	9	(56%)
−	70				17	68	**(25%)**	4	64	**(6%)**	29	68	(43%)
Ct history													
+	21				17	21	**(81%)**	7	21	**(33%)**	14	21	**(67%)**
−	56				8	56	**(14%)**	1	56	**(2%)**	20	56	**(36%)**

Percentages in bold indicate significant differences in proportion positive for that determinant.

**Table 2:** Comparison of the sensitivity, specificity, and agreement between test results of different antibodies in serum and swab with active or past Chlamydia infection or complications thereof, in fertility clinic and STI clinic.

	Sensitivity	Specificity	Kappa-value
Fertility clinic Probable past Ct infection^#^, PID, or tubal pathology reported			
Serum-IgG	79%	89%	0.67 (*P* < 0.001)
Swab-IgG	63%	64%	0.23 (*P* = 0.03)
Swab-IgA	29%	98%	0.34 (*P* < 0.001)
Tubal pathology			
Serum-IgG	89%	75%	0.36 (*P* < 0.001)
Swab-IgG	56%	57%	0.06 (*P* = 0.47)
Swab-IgA	56%	94%	0.41 (*P* < 0.001)
STI clinic Active infection*/probable past Ct infection^#^			
Serum-IgG	87%	95%	0.83 (*P* < 0.001)
Serum-IgA	32%	95%	0.32 (*P* < 0.001)
Swab-IgG	43%	63%	0.06 (*P* = 0.53)
Swab-IgA	38%	90%	0.32 (*P* < 0.001)

*Active infection: PCR positive at STI clinic.

^#^Probable past Chlamydia infection. #: history of STI at STI clinic or reported Chlamydia infection in the past or complications related to chlamydia (PID, tubal pathology) in fertility clinic.
